# Cyclophosphamide Regulates N6-Methyladenosine and m6A RNA Enzyme Levels in Human Granulosa Cells and in Ovaries of a Premature Ovarian Aging Mouse Model

**DOI:** 10.3389/fendo.2019.00415

**Published:** 2019-06-27

**Authors:** Boxian Huang, Chenyue Ding, Qinyan Zou, Wei Wang, Hong Li

**Affiliations:** ^1^Center of Reproduction and Genetics, Suzhou Municipal Hospital, Affiliated Suzhou Hospital of Nanjing Medical University, Suzhou, China; ^2^State Key Laboratory of Reproductive Medicine, Nanjing Medical University, Nanjing, China

**Keywords:** cyclophosphamide, m^6^A, RNA methyltransferase, RNA demethylase, RNA effector

## Abstract

Cyclophosphamide (CTX) is one of the most frequently used alkylating anticancer drugs. CTX is associated with reproductive failure and premature ovarian insufficiency (POI) or premature ovarian aging. Much less is known about the mechanism by which CTX affects female fertility through N6-methyladenosine (m^6^A) levels. In this case-controlled study, we employed human ovarian granulosa cells and mice as experimental models *in vitro* and *in vivo*. m^6^A test kit was developed to determine the content in RNA, and qPCR and western blot were used to examine the expression levels of RNA methyltransferases, demethylases, and effectors. Results showed that CTX increased the m^6^A level in a time- and concentration-dependent manner. The expression levels of RNA methyltransferases were significantly higher in the CTX treatment group than in the control group with time and concentration dependence, except for RBM15 and WTAP. CTX significantly inhibited the expression levels of RNA demethylase FTO in a time- and concentration-dependent manner but not ALKBH5. The expression levels of RNA effectors were reduced by CTX in a time- and concentration-dependent manner. These data suggest that CTX increased the expression levels of m^6^A and may be responsible for the increase in RNA methyltransferases and decrease in RNA demethylases in a time- and concentration-dependent manner.

## Introduction

Premature ovarian failure (premature ovarian aging) and infertility are serious side effects of chemotherapy treatments in young female cancer patients (1). Cyclophosphamide (CTX) belongs to the alkylating category of chemotherapy drugs, the most destructive among all types of chemotherapy drugs (2). Of all the chemotherapy drug classes, it is highly toxic to the ovaries and carries the greatest risk of premature ovarian failure (3). CTX can act on primordial follicles, decreasing the ovarian reserve, resulting in premature ovarian failure and inducing genomic and organelle damage in the cells regardless of the stage of cell cycle, leading to more widespread apoptosis and organ damage (4, 5). CTX is also commonly used in the treatment of a variety of cancer diseases and hematological malignancies, as well as chronic, non-malignant diseases such as systemic lupus erythematosus (6). The fertility damage to ovaries from some anticancer therapies is irreversible. To develop a pharmacological means of attenuating this destruction of the ovarian reserve, it is vital to understand the mechanisms involved in chemotherapy-induced primordial follicle loss.

Until now, more than one hundred different RNA modifications were known in mRNA and non-coding RNAs, among which N6-methyladenosine (m^6^A) is the most prevalent mRNA modification in eukaryotes (7). Globally, m^6^A is enriched around stop codons, in 3' untranslated regions (3' UTRs) and within long internal exons, and m^6^A occurs more in precursor mRNAs (pre-mRNAs) (8). The m^6^A modification is preferentially embedded within the consensus sequence motif composed of 5′-RRACU-3′ (R = A or G) (7). m^6^A regulates gene expression, RNA metabolic processes, mRNA stability, translation efficiency, alternative splicing, and cytoplasmic mRNA turnover (9).

In addition to the core complex, a number of other proteins have been implicated in regulating m^6^A: RNA methyltransferase (writer), demethylase (eraser) and effector (reader) (10). RNA m^6^A methylated modification is mediated by a core complex of three components: methyltransferase-like 3 (METTL3), METTL14, and Wilms' tumor 1-associating protein (WTAP) (11). Formation of m^6^A is catalyzed by the activity of METTL3, which physically interacts with METTL14, WTAP, Virlike m^6^A methyltransferase-associated (KIAA1429/VIRMA), and RNA-binding motif 15 (RBM15) (12). Oocyte maturation is a complex process that including germinal vesicle, germinal vesicle breakdown, metaphase-I, and metaphase-II stage. Previous study indicated that highly methylated or hypomethylated mRNAs can affect cell division at meiotic stage (13). m^6^A is a reversible modification, and two m^6^A demethylases have been identified: fat-mass and obesity-associated protein (FTO) and α-ketoglutarate-dependent dioxygenase alkB homolog 5 (ALKBH5). Knockdown of FTO in human ovary granulosa cells inhibited cell growth and caused apoptosis (14). The functional consequence of m^6^A marks on RNAs is mediated by a “reader” protein family, members of which carry a YT521-B homology (YTH) domain: YTHDF1, 2, 3, and YTHDC1. Knockout of YTHDC1 inhibited embryo viability and germline development in mice (15). As we know, CTX can inhibit the ovarian function by inducing DNA damage in oocyte. Besides, RNA m6A modification induce DNA damage response by the enzyme of METTL3 and FTO (16). However, little is known about whether CTX affects female reproductive ability by affecting the level of m^6^A modification.

To answer this question, we designed an experimental methodology combining *in vivo* and *in vitro* models of ovary and granulosa cells from humans and mice, respectively, with two specific aims: (i) to determine whether CTX affects m^6^A levels in *in vivo* and *in vitro* models; (ii) to determine whether CTX changes the level of m^6^A by affecting RNA methylation regulators. Our results showed that CTX increased the m^6^A level and affected the expression levels of RNA methylation regulators in a time- and concentration-dependent manner.

## Materials and Methods

### Acquisition and Cultivation of Primary Human Ovarian Granulosa Cells (Hgcs) From Patients

Normal hGCs were obtained from patients (ages < 35) with tubal occlusion. The exclusion criteria is that the women with previous radiotherapy, ovarian surgery, known abnormal karyotype or autoimmune diseases. Ethics approval information and informed consent from patients were obtained (3). Recombinant FSH (Puregon; Schering Plough, New Jersey, USA) and GnRH antagonist (Merck, Frosst, Montreal, Canada) were employed to treat all subjects. Follicular development was monitored by vaginal ultrasound examinations. Ten thousand International Unit of human chorionic gonadotropin (hCG) (Pregnyl, Merck) was used to induce maturation of follicle. During the process of oocyte retrieval, follicular fluid was collected. Purified hGCs were acquired by density centrifugation method as previously described (3). Culture medium for primary hGCs was formed, that included DMEM/F12 media (Thermo, USA), 1% penicillin/streptomycin, 100 mg/ml streptomycin sulfate (Thermo, USA), 1X GlutaMAX (Thermo, USA), and 10% fetal bovine serum (complete medium). CTX (Sigma, USA) was used at different doses (20, 40, and 60 μg/ml) for treating hGCs, CTX with 60 μg/ml was used at different time point, respectively (0, 3, 6, 9, and 12 days). In all of the experiment, the culture medium was changed every other day, the information of patients were listed in Supplemental Table 2.

### Establishment of POI Mouse Model

According to the institutional guidelines that from Nanjing Medical University with Institutional Animal Care and Use Committee approval, female ICR mice with 7 or 8 weeks of age were obtained. To generate the mouse model, according to our previous methods, CTX (Sigma, USA) was used at different doses (40, 80, and 120 mg/kg), CTX with 120 mg/kg was used at different time point, respectively (0, 1, 2, 4, and 8 weeks) (14). The animals were divided into four groups: control (saline injection) and 40, 80, 120 mg/kg CTX treatment groups (*n* = 10 per group).

### Measurement of m^6^A RNA Methylation

The global m^6^A levels in mRNA were determined in 200-ng aliquots of mRNA extracted from hGCs or mouse ovary using an EpiQuik m^6^A RNA Methylation Quantification Kit (cat. no. P-9005; Epigentek, Farmingdale, NY, USA) according to the manufacturer's instructions. Briefly, a standard curve was prepared by positive control at the six different concentrations: 0.01, 0.02, 0.05, 0.1, 0.2, and 0.5 ng/μl. Equal volumes of RNA solution (8 μL) and negative control were added to the strip wells. The plate was covered with a plate seal and incubated at 37°C for 90 min. Afterward, the capture antibody diluted 1:1,000 was added to the wells after three times washing with 150 μl of washing buffer (WB) and incubated at room temperature for 1 h. The detection antibody (1:2,000 dilution) was then added to the wells after washing three times. The enhancer solution was added to each well and incubated at room temperature for 30 min. After washing three times with 150 μl of WB, the detection solution was added to each well and incubated at room temperature for 10 min away from light. Thereafter, stop solution was added to stop the enzyme reaction with 100 μl. The absorbance was read on a microplate reader at 450 nm.

$$\begin{array}{l} {\text{m}}^{6}\text{A}\left(\text{ng}\right)=\left(\text{OD }:\text{ sample }-\text{ OD }:\text{ background}\right)\\ \;/\text{slope of standard curve}\\ {\text{m}}^{6}\text{A}\left(\text{\%}\right)={\text{m}}^{6}\text{A amount }\left(\text{ng}\right)/200\text{ ng}. \end{array}$$

### RNA Extraction and QRT-PCR

Total RNA from the hGCs and mouse ovarian tissues treated with different concentrations of CTX at 0, 3, 6, 9, and 12 days or 0, 1, 2, 4, and 8 weeks, respectively, were extracted using the QIAGEN RNeasy Mini Kit (QIAGEN) according to manufacturer instructions. For mRNA analysis, reverse-transcribed to cDNA was performed with the PrimeScript RT Reagent Kit (Takara, Japan) according to the manufacturer's methods. Quantitative real-time PCR was performed on an Applied Biosystems 7900 Fast Real Time PCR System with SYBR Premix Ex Taq (Takara, Japan). The cycle time (Ct) values were assessed with the Sequence Detection System and analysis software (Applied Biosystems). Relative expression levels of targets were determined using the comparative 2^−ΔΔ*Ct*^ method with *GAPDH* as internal control. The experiments were executed three times, and *p* < 0.05 was considered to be a significant. The mRNA expression levels were calculated after compared with internal control (GAPDH), and then all of these values were compared with the control group. All primers were listed in Supplemental Table 1.

### Western Blot Analysis

The hGCs and mouse ovarian tissues treated with different concentrations of CTX at 0, 3, 6, 9, and 12 days or 0, 1, 2, 4, and 8 weeks, respectively were harvested and dissociated in a lysis buffer. Protein from each sample was extracted and quantified using a BCA Protein Assay kit (Beyotime, China). The proteins were loaded onto 10% gels and separated using SDS-PAGE (sodium dodecyl sulfate polyacrylamide gel electrophoresis). Next, the separated proteins were transferred to polyvinylidene difluoride (PVDF) membranes (Millipore, USA). Membranes were blocked for 1 h at room temperature in 5% milk in TBS-T and then were incubated with the primary antibodies from ABCAM company in USA (anti-human) overnight at 4°C, respectively are, METTL3 (Cat: ab195352), METTL14 (Cat: ab98166), WTAP (Cat: ab195380), FTO (Cat: ab92821), KIAA1429 (Cat: ab178966), RBM15 (Cat: ab244374), ZC3H13 (Cat: ab70802), ALKBH5 (Cat: ab195377), YTHDF1 (Cat: ab220162), YTHDF2 (Cat: ab245129), YTHDF3 (Cat: ab83716), YTHDC1 (Cat: ab220159), GAPDH (Cat: ab8245), and β-Tubulin (Cat: ab7291), and they were subsequently incubated with the appropriate secondary antibodies (goat anti-rabbit HRP conjugates; Jackson Immunoresearch, West Grove, USA). The specific signals were detected with an enhanced chemiluminescence reagent (Pierce ECL Western blotting Substrate, Thermo). Finally, the protein bands were detected using a chemiluminescence detection system (Tanon, China), and the protein density of each band was analyzed using the Image J software (National Institutes of Health, USA). The protein expression levels were calculated after compared with internal control (GAPDH or β-Tubulin), and then all of these values were compared with the control group. The experiments were executed three times, *p* < 0.05 was considered to be a statistically significant difference.

### m^6^A Dot Blot Assay

Total RNA was isolated immediately after the cells were harvested using Trizol (Invitrogen, 15596-018) according to the manufacturer's instructions; mRNA was prepared from total RNA using the Dynabeads mRNA purification kit (Ambion, catalog no. 61006). The concentration of purified mRNA was determined with a NanoDrop. RNA samples were immobilized twice using UV cross-linking (Stratalinker 2400) and were incubated at 80°C for 1 h. The blot was blocked using blocking buffer (PBS, 10% SDS, 1 mM EDTA) for 20 min. The mRNA was used for dot blot analysis using an antibody specific for m^6^A (1:1,000; Synaptic Systems; catalog no. 202003). The membrane was incubated with HRP-conjugated Goat anti-rabbit IgG (DakoCytomation, p0448, 1:10,000 dilution; 20 ng/ml) in 10 ml of antibody dilution buffer for 1 h at room temperature. The membrane was washed four times for 10 min each in 10 ml of wash buffer. To visualize the spots, the membrane was incubated with 3 ml of ECF substrate (GE Healthcare) for 5 min in darkness at room temperature. The intensity of the dot blot signal was analyzed by Image J software.

### Statistical Analysis

All results are shown as the means ± SD. Statistically significant differences were determined by two-way ANOVA with SPSS 17.0 software, and *p* < 0.05 was regarded as statistically significant.

## Results

### CTX Increased m^6^A Levels in a Concentration- and Time-Dependent Manner

**Figure 1:**
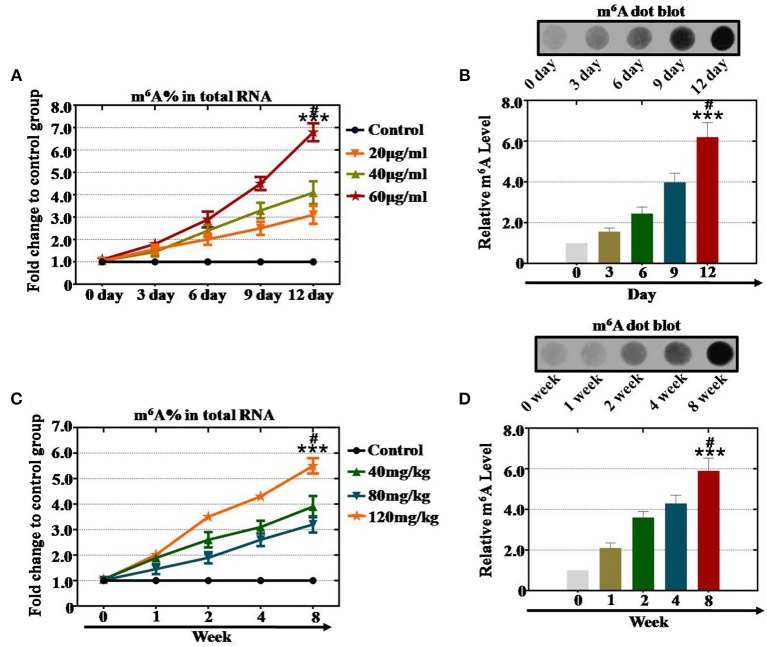
Quantification and statistical analysis of the m^6^A expression levels after CTX treatment *in vivo* and *in vitro*. **(A)** The m^6^A content was tested in hGCs by a m^6^A test kit after treatment with 20, 40, and 60 μg/ml CTX from 0 to 12 days. **(B)** The dot blot method tested the m^6^A levels in hGCs after treatment with 60 μg/ml CTX from 0 to 12 days. **(C)** The m^6^A content was tested in mouse ovaries by a m^6^A test kit after treatment with 40, 80, and 120 mg/kg CTX from 0 to 8 weeks. **(D)** The dot blot method detected the m^6^A levels in mouse ovaries after treatment with 40, 80, and 120 mg/kg CTX from 0 to 8 weeks. All experiments were performed three times. The error bars indicate SD; ***p < 0.001 (compared with the 0 day or 0 week group); ^#^*p* < 0.05 (compared with 9 days or 4 weeks in same CTX concentration group).

**Figure 2:**
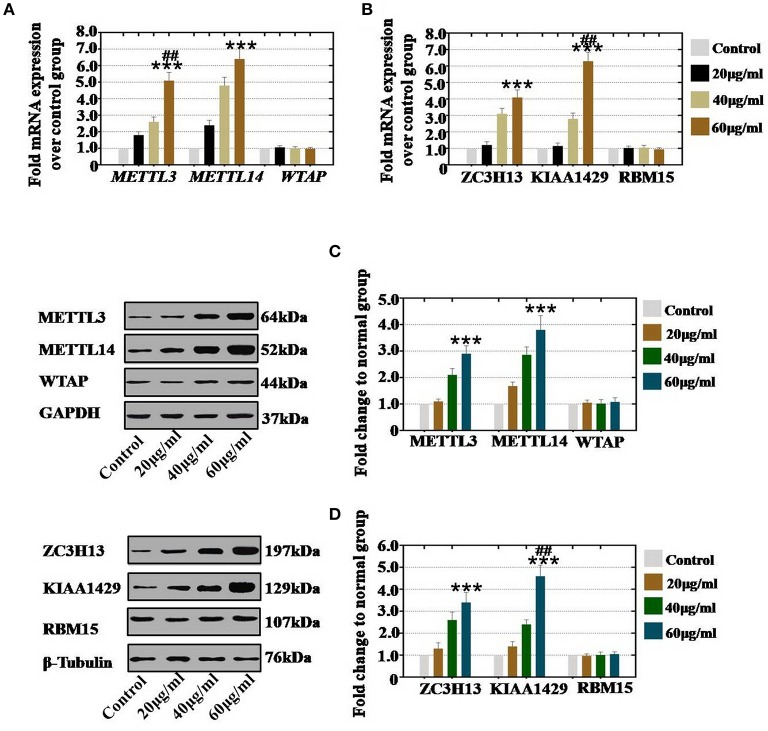
Quantification and concentration-dependence analysis of the RNA methyltransferases expression levels after CTX treatment *in vivo*. **(A)** The mRNA expression levels of METTL3, METTL14 and WTAP in hGCs were measured after treatment with CTX (20, 40, and 60 μg/ml). **(B)** The mRNA expression levels of ZC3H13, KIAA1429, and RBM15 in hGCs were measured after treatment with CTX (20, 40, and 60 μg/ml). **(C)** The protein expression levels of METTL3, METTL14 and WTAP in hGCs were measured after treatment with CTX (20, 40, and 60 μg/ml). **(D)** The protein expression levels of ZC3H13, KIAA1429, and RBM15 in hGCs were measured after treatment with CTX (20, 40, and 60 μg/ml). All experiments were performed three times. The error bars indicate SD; ***p < 0.001 (compared with control group); ^*##*^*p* < 0.01 (compared with the 40 μg/ml CTX concentration group).

To investigate the relationship between m^6^A levels and CTX in the female reproductive system, in our previous study (14), we already collected and examined ovarian tissue and hGCs in our reproductive center. *In vitro* study results exhibited that the level of m^6^A was elevated significantly (310, 410, and 680%) in the 20, 40 and 60 μg/ml CTX groups, respectively, compared with the control group after treatment for 12 days (Figure 1A). Our results also indicated that CTX induced a high level of m^6^A in a time-dependent manner. After CTX treatment at 6, 9, and 12 days, 60 μg/ml CTX increased the level of m^6^A gradually to 290, 450, and 680%, respectively, compared to the control group (Figure 1A). CTX (40 μg/ml) also elevated the level of m^6^A to 240, 330, and 410% at 6, 9, and 12 days, respectively, relative to the control group (Figure 1A). Meanwhile, as the treatment time increased from 0 to 12 days, the 20 and 40 μg/ml CTX treatment elevated the m^6^A <60 μg/ml CTX treatment (Figure 1A). Moreover, the dot blot assay showed the same results that 60 μg/ml CTX increased the m^6^A levels to 210, 360, 430, and 590% at 3, 6, 9, and 12 days, respectively, compared to the levels of the control group (Figure 2B).

To confirm the relationship between the m^6^A level and CTX at a deeper level, the mouse ovaries were treated with different concentrations of CTX as reported in our previous research (14). An m^6^A test kit and dot blot analyses were used to assess the relationship between m^6^A and CTX. First, the m^6^A test kit assay revealed that CTX increased the level of m^6^A in a concentration-dependent manner. The m^6^A level was raised to 320, 390, and 550% after CTX treatment with 40, 80, and 120 mg/kg at 8 weeks compared to the levels of the control group (0 week) (Figure 1C). Furthermore, our results also found that CTX induced high level m^6^A in a time-dependent manner in an *in vivo* study. After 120 mg/kg CTX treatment at 2, 4 and 8 weeks, the level of m^6^A was increased to 350, 430, and 550% compared to the control group (Figure 1C). CTX (80 mg/kg) also elevated the level of m^6^A to 260, 310, and 390% at 2, 4, and 8 weeks, respectively, relative to the control group (Figure 1C). In addition, after 120 mg/kg CTX treatment, the dot blot assay showed that the level of m^6^A was raised significantly to 245, 398, and 620% at 2, 4, and 8 weeks, respectively, relative to that of the control group (Figure 1D).

As a whole, we found that the increased level of m^6^A was caused by CTX treatment in a time- and concentration-dependent manner.

### CTX Elevated the Level of RNA Methyltransferases in a Concentration-Dependent Manner

To confirm whether the concentration of CTX affects the expression of RNA methyltransferases, hGCs, and mouse ovaries were collected after treatment with different concentrations of CTX. *In vitro* study, our results showed that CTX treatment at in 20, 40, and 60 μg/ml significantly increased the mRNA expression levels of METTL3 and METTL14 to 180, 260, and 510% and to 240, 480, and 640%, respectively, relative to the expression levels of the control group (Figure 2A). CTX treatment at 20, 40, and 60 μg/ml significantly increased the mRNA expression levels of ZC3H13 and KIAA1429 to 121, 310, and 410% and to 115, 280, and 630%, respectively, relative to the expression levels of the control group (Figure 2B). Moreover, western blot assay revealed that the protein expression level of METTL3 (110, 210, and 290%) and METTL14 (168, 286, and 380%) were raised gradually in 20, 40, 60 μg/ml CTX treatment groups, respectively, relative to the expression level in the control group (Figure 2C). CTX elevated the protein expression level of ZC3H13 and KIAA1429 to 130, 260, and 340% and 140, 240, and 460% in a CTX concentration-dependent manner (20, 40, and 60 μg/ml, respectively) relative to the expression levels of the control group (Figure 2D). However, the gene and protein expression levels of WTAP and RBM15 were not changed in different CTX concentration treatment groups.

**Figure 3:**
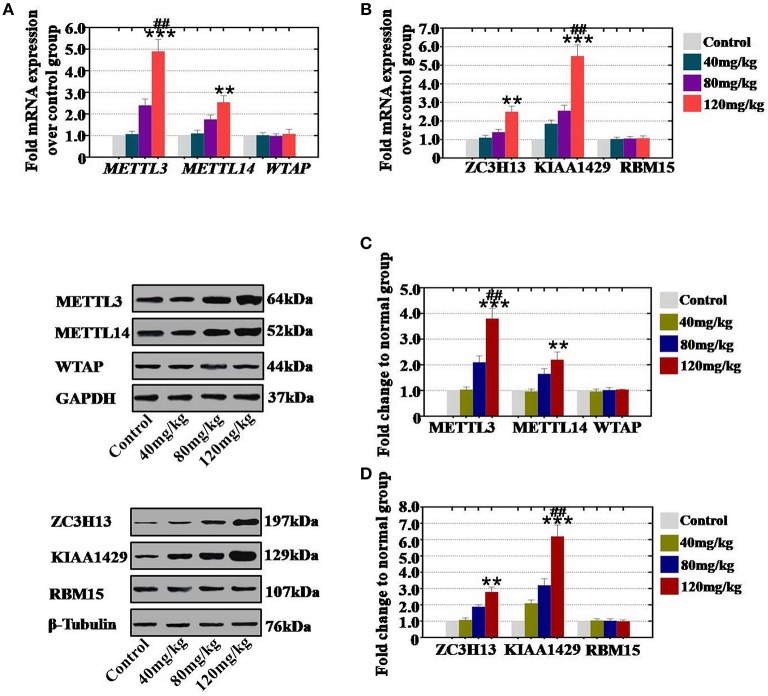
Quantification and concentration-dependence analysis of the RNA methyltransferase expression levels after CTX treatment *in vitro*. **(A)** The mRNA expression levels of METTL3, METTL14, and WTAP in mouse ovary were measured after treatment with CTX (40, 80, and 120 mg/kg). **(B)** The mRNA expression levels of ZC3H13, KIAA1429, and RBM15 in mouse ovary were measured after treatment with CTX (40, 80, and 120 mg/kg). **(C)** The protein expression levels of METTL3, METTL14, and WTAP in mouse ovary were measured after treatment with CTX (40, 80, and 120 mg/kg). **(D)** The protein expression levels of ZC3H13, KIAA1429 and RBM15 in mouse ovary were measured after treatment with CTX (40, 80, and 120 mg/kg). All experiments were performed three times. The error bars indicate SD; **p < 0.01; ***p < 0.001 (compared with the control group); ^*##*^*p* < 0.01 (compared with the 80 mg/kg CTX concentration group).

*In vivo* study, our results demonstrated that our results showed that CTX significantly increased the mRNA expression level of METTL3 and METTL14 to 107, 240, and 490%, and to 110, 175, and 255% in 40, 80, and 120 mg/kg treatment groups, respectively, relative to the levels of expression in the control group (Figure 3A). CTX (40, 80, and 120 mg/kg treatments) increased the mRNA expression level of ZC3H13 and KIAA1429 to 110, 140, and 250% and to 185, 255, and 550%, respectively, relative to the levels of the control group (Figure 3B). Moreover, western blot assay revealed that the protein expression level of METTL3 (104, 210, and 380%) and METTL14 (97, 165, and 220%) were raised gradually in the 40, 80, 120 mg/kg CTX treatment groups, respectively, relative to the control group (Figure 3C). CTX (40, 80 and 120 mg/kg) elevated the protein expression level of ZC3H13 and KIAA1429 to 108, 189, and 280%, and to 210, 320, and 620% in a concentration-dependent manner relative to the levels of expression of the control group (Figure 3D). However, the gene and protein expression levels of WTAP and RBM15 were not changed in different CTX concentration treatment groups.

Thus, we found that the increased level of RNA methyltransferases (METTL3 and METTL14) were caused by CTX *in vivo* and *in vitro* and that these increases in expression were CTX concentration-dependent.

### CTX Elevated the Level of RNA Methyltransferases in a Time-Dependent Manner

**Figure 4:**
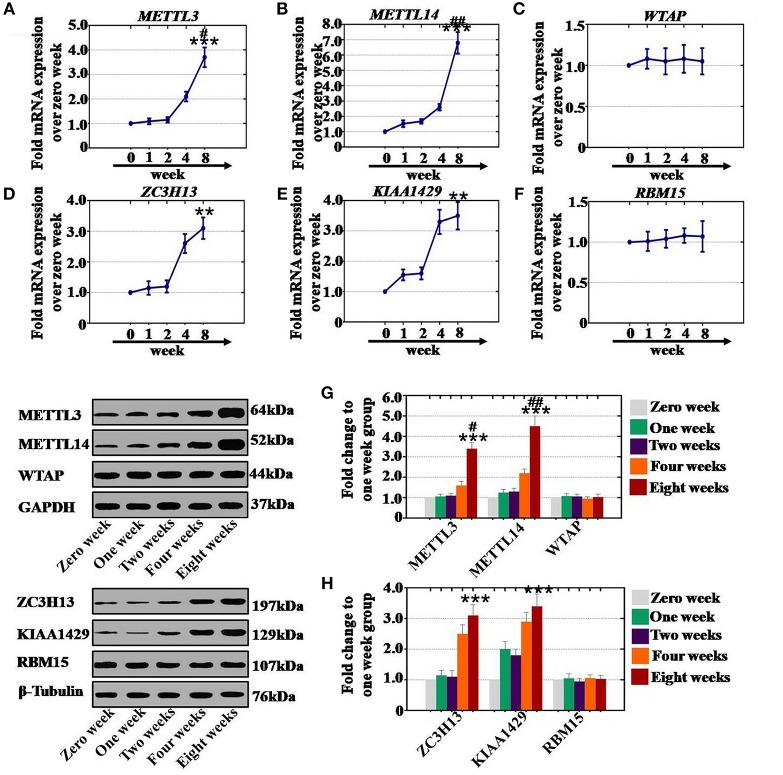
Quantification and time-dependence analysis of the RNA methyltransferase expression levels after CTX treatment *in vivo*. **(A–F)** The mRNA expression levels of METTL3, METTL14, WTAP, ZC3H13, KIAA1429, and RBM15 in human ovary were tested after treatment with 120 mg/kg CTX from 0 to 8 weeks. **(G)** The western blot method determined the expression levels of METTL3, METTL14, and WTAP in human ovary after treatment with 120 mg/kg CTX from 0 to 8 weeks. **(H)** The western blot method determined the expression levels of ZC3H13, KIAA1429 and RBM15 in human ovary after treatment with 120 mg/kg CTX from 0 to 8 weeks. All experiments were performed three times. The error bars indicate SD; **p < 0.01; ***p < 0.001 (compared with the control group); ^#^*p* < 0.05; ^*##*^*p* < 0.01 (compared with the four weeks group).

To confirm whether CTX affects the expression of RNA methyltransferases in a time-dependent manner, after CTX treatment, ovaries were collected from mice at different time points. Our results revealed that the mRNA expression levels of METTL3 (210 and 370%), METTL14 (260 and 680%), ZC3H13 (260 and 310%), and KIAA1429 (330 and 350%) were significantly higher at 4 and 8 weeks than those expression levels in the control group (0 week) (Figures 4A,B,D,E). There were not any significant changes in METTL3, METTl14, ZC3H13, or KIAA1429 expression at 1 or 2 weeks (Figures 4A,B,D,E). Furthermore, CTX did not greatly affect the gene expression levels of WTAP or RBM15 at different time points (Figures 4C,F). In addition, western blot assay results demonstrated that the protein expression levels of METTL3 (160 and 340%) and METTL14 (220 and 450%) were raised gradually at 4 and 8 weeks, respectively, relative to the expression levels of the control group (0 week) (Figure 4G). CTX enhanced the protein expression levels of ZC3H13 and KIAA1429 to 250 and 310%, and to 290 and 340% in a time-dependent manner at 4 and 8 weeks relative to the control group (Figure 4H). There were no significant changes of METTL3, METTl14, ZC3H13, and KIAA1429 at protein expression levels at 1 and 2 weeks (Figures 4G,H). Furthermore, CTX did not greatly affect the protein expression levels of WTAP or RBM15 at different time points (Figures 4G,H).

As a whole, CTX increased the mRNA and protein expression levels of RNA methyltransferases in a time-dependent manner.

### CTX Decreased the Level of RNA Demethylases in a Concentration- and Time-Dependent Manner

**Figure 5:**
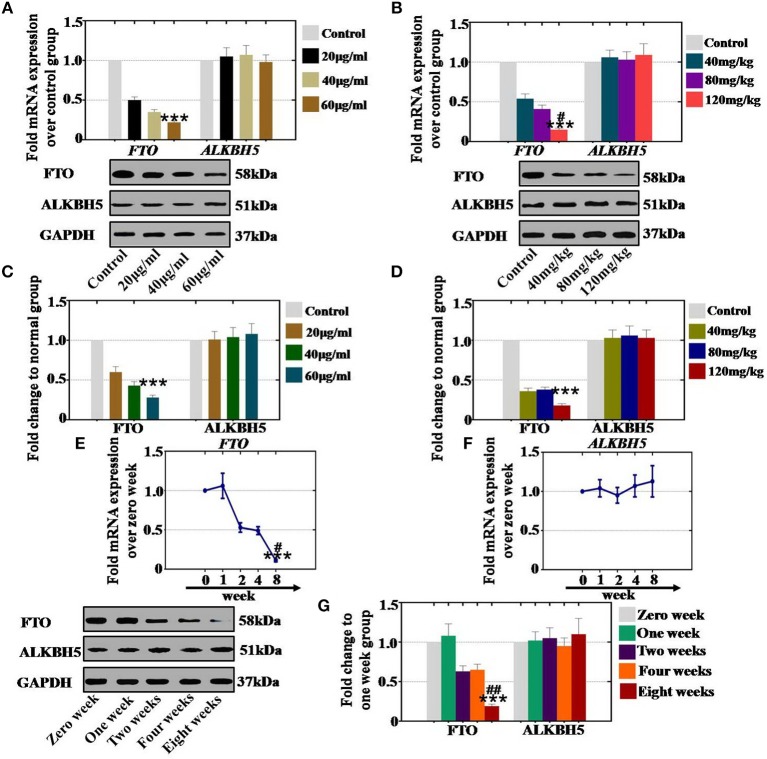
Quantification, concentration- and time-dependence analysis of the RNA demethylase expression levels after CTX treatment *in vivo* and *in vitro*. **(A)** The mRNA expression levels of FTO and ALKBH5 in hGCs were determined after treatment with CTX at concentrations of 20, 40, and 60 μg/ml. **(B)** The mRNA expression levels of FTO and ALKBH5 in mouse ovary were determined after treatment with 40, 80, and 120 mg/kg CTX. **(C)** The protein expression levels of FTO and ALKBH5 in hGCs were determined after treatment with CTX (20, 40, and 60 μg/ml). **(D)** The protein expression levels of FTO and ALKBH5 in mouse ovary were determined after treatment with 40, 80, 120 mg/kg CTX. **(E)** The mRNA expression levels of FTO and ALKBH5 in hGCs were determined after treatment with 60 μg/ml CTX from 0 to 8 weeks. **(F)** The mRNA expression levels of FTO and ALKBH5 in hGCs were tested after treatment with 120 mg/kg CTX from 0 to 8 weeks. **(G)** The protein expression levels of FTO and ALKBH5 in mouse ovary were tested after treatment with 120 mg/kg CTX from 0 to 8 weeks. The error bars indicate SD; ***p < 0.001 (compared with the control group); ^#^*p* < 0.05; ^*##*^*p* < 0.01 [compared with the 80 mg/kg CTX concentration group in **(B)**, compared with the four weeks group in **(E,G)**].

To confirm the relationship between the expression level RNA demethylases and CTX, hGCs and ovaries were collected and assessed with qPCR and western blot analyses. *In vitro* and *in vivo* study, the qPCR assay results revealed that the expression of FTO was significantly inhibited to 50, 35, and 22% (with 20, 40 and 60 μg/ml, respectively) and to 54, 41, and 15% (with 40, 80, and 120 mg/kg, respectively) in different CTX concentration treatment groups relative to the control group (Figures 5A,B). Western blot assays showed similar results. Protein level tests indicated that the level of FTO was gradually higher (60, 43, and 28% *in vitro* and 36, 38, and 18% *in vivo*) in different CTX concentration treatment groups (20, 40 and 60μg/ml *in vitro* and 40, 80 and 120 mg/kg *in vivo*) than in the control group (Figures 5C,D). However, RNA demethylase ALKBH5 did not any change *in vivo* or *in vitro* after CTX treatment at different concentrations.

Additionally, in order to confirm the time-dependence, mouse ovaries from 0, 1, 2, 4, and 8 weeks were collected. Our results demonstrated that CTX inhibited the mRNA expression levels of FTO to 53, 49, and 11% corresponding to CTX treatment time points at 2, 4, and 8 weeks relative to the level of expression of the control group (0 week) (Figure 5E). Moreover, the protein expression level of FTO was decreased to 63, 65, and 19% after CTX treatment at 2, 4, and 8 weeks, respectively, compared to the levels of the control group (Figure 5G). ALKBH5 mRNA and protein levels were not changed with CTX treatment (Figures 5F,G).

In summary, CTX decreased the mRNA and protein expression levels of RNA demethylase FTO in a concentration- and time-dependent manner.

### CTX Inhibited the Level of RNA Effectors in a Concentration-Dependent Manner

**Figure 6:**
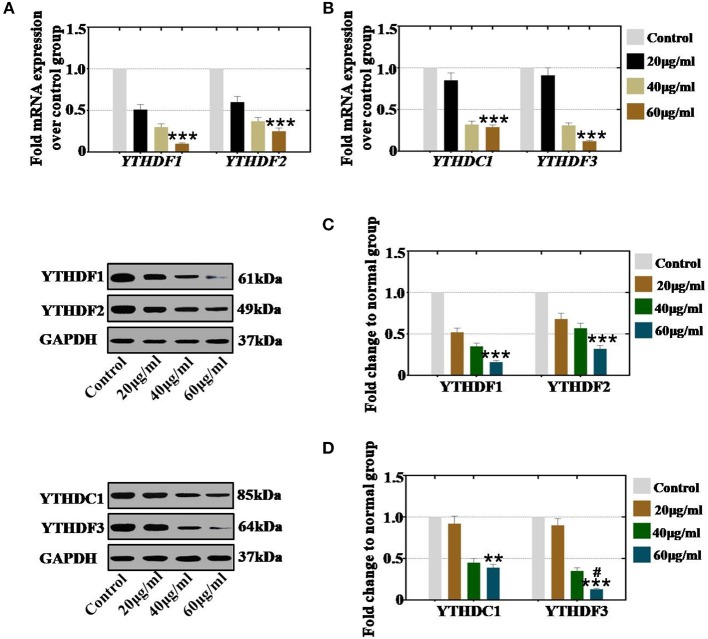
Quantification and concentration-dependence analysis of the RNA effector expression levels after CTX treatment *in vivo*. **(A)** The mRNA expression levels of YTHDF1 and YTHDF2 in hGCs were measured after CTX treatment (20, 40, and 60 μg/ml). **(B)** The mRNA expression levels of YTHDC1 and YTHDF3 in hGCs were measured after treatment with CTX (20, 40, and 60 μg/ml). **(C)** The protein expression levels of YTHDF1 and YTHDF2 in hGCs were measured after treatment with CTX (20, 40, and 60 μg/ml). **(D)** The protein expression levels of YTHDC1 and YTHDF3 in hGCs were measured after treatment with CTX (20, 40, and 60 μg/ml). All experiments were performed three times. The error bars indicate SD; **p < 0.01; ***p < 0.001 (compared with control group); ^#^*p* < 0.05 (compared with the 40 μg/ml CTX concentration group).

To confirm whether CTX affects the expression of RNA effectors in a concentration-dependent manner, hGCs and mouse ovaries were collected after treatment with different concentrations of CTX. *In vitro* study, our results showed that CTX (20, 40, and 60 μg/ml) significantly decreased the mRNA expression level of YTHDF1 and YTHDF2 to 51, 30, and 10% and to 60, 37, and 25% relative to the levels of the control group (Figure 6A). CTX (20, 40, and 60 μg/ml) significantly decreased the mRNA expression levels of YTHDC1 and YTHDF3 to 85, 32, and 29% and to 91, 31, and 12% relative to the control group (Figure 6B). Moreover, western blot analysis revealed that the protein expression levels of YTHDF1 (52, 35, and 16%) and YTHDF2 (68, 57, and 3%) were reduced gradually in 20, 40, and 60 μg/ml treatment groups, respectively, relative to the levels of the control group (Figure 6C). CTX inhibited the protein expression levels of YTHDC1 and YTHDF3 to 92, 45, and 39% and to 90, 35, and 13% following different CTX treatment concentrations (20, 40, and 60 μg/ml) relative to the levels of the control group (Figure 6D).

**Figure 7:**
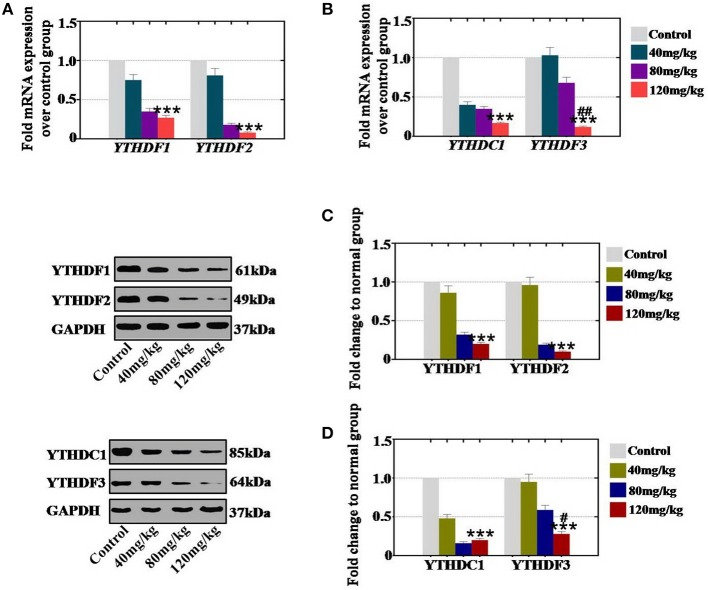
Quantification and concentration-dependence analysis of the RNA effector expression levels after CTX treatment *in vitro*. **(A)** The mRNA expression levels of YTHDF1 and YTHDF2 in mouse ovary were measured after treatment with CTX (40, 80, and 120 mg/kg). **(B)** The mRNA expression levels of YTHDC1 and YTHDF3 in mouse ovary were measured after treatment with CTX (40, 80, and 120 mg/kg). **(C)** The protein expression levels of YTHDF1 and YTHDF2 in mouse ovary were measured after treatment with CTX with (40, 80, and 120 mg/kg). **(D)** The protein expression levels of YTHDC1 and YTHDF3 in mouse ovary were measured after treatment with CTX (40, 80, and 120 mg/kg). All experiments were performed three times. The error bars indicate SD; ***p < 0.001 (compared with the control group); ^#^*p* < 0.05; ^*##*^*p* < 0.01 (compared with the 80 mg/kg CTX concentration group).

In an *in vivo* study, our results demonstrated that CTX significantly reduced the mRNA expression levels of YTHDF1 and YTHDF2 to 75, 35, and 27% and to 81, 18, and 8% in the 40, 80, and 120 mg/kg treatment groups, respectively, relative to the expression level of the control group (Figure 7A). The mRNA expression levels of YTHDC1 and YTHDF3 were decreased to 40, 35, and 17% and to 103, 68, and 12% in the 40, 80, and 120 mg/kg CTX treatment groupsrelative to the expression levels of the control group (Figure 7B). Moreover, western blot assay revealed that the protein expression levels of YTHDF1 (86, 32, and 20%) and YTHDF2 (96, 19, and 10%) were decreased gradually in the 40, 80, and 120 mg/kg treatment groups, respectively, relative to the expression levels of the control group (Figure 7C). CTX inhibited the protein expression levels of YTHDC1 and YTHDF3 to 48, 16, and 20% and to 95, 59, and 28% at different CTX treatment concentrations (40, 80, and 120 mg/kg, respectively) relative to the expression levels of the control group (Figure 7D).

Thus, we found that the decreased levels of RNA effectors were caused by CTX in a concentration-dependent manner both *in vivo* and *in vitro*.

### CTX Inhibited the Level of RNA Effectors in a Time-Dependent Manner

**Figure 8:**
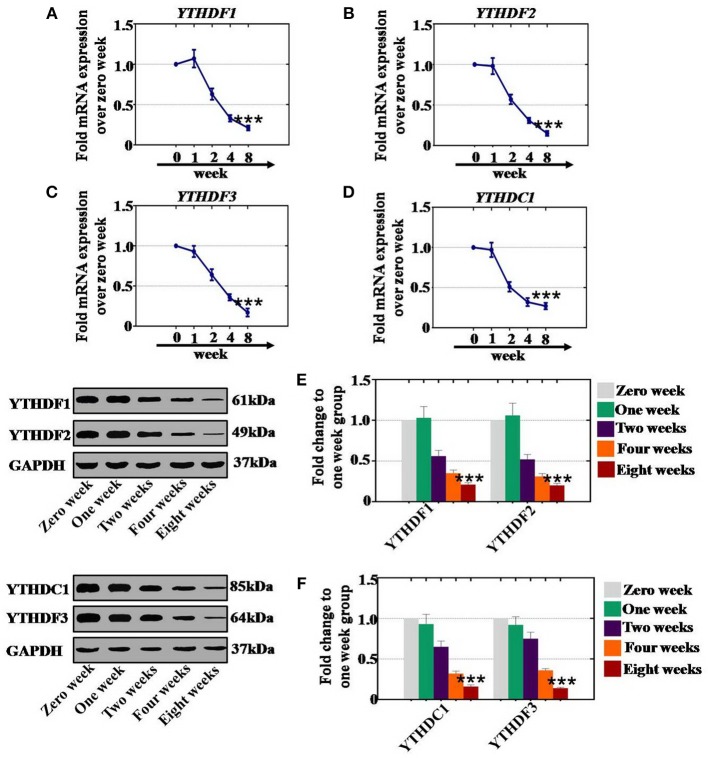
Quantification and time-dependence analysis of the RNA effector expression levels after CTX treatment *in vivo*. **(A–D)** The mRNA expression levels of YTHDF1, YTHDF2, YTHDC1, and YTHDF3 in human. **(A–D)** Ovary were tested after treatment with 120 mg/kg CTX from 0 to 8 weeks. **(E)** The western blot method determined the expression levels of YTHDF1 and YTHDF2 in human ovary after treatment with 120 mg/kg CTX from 0 to 8 weeks. **(F)** The western blot method determined the expression levels of YTHDC1 and YTHDF3 in human ovary after treatment with 120 mg/kg CTX from 0 to 8 weeks. All experiments were performed three times. The error bars indicate SD; ***p < 0.001 (compared with the control group).

To confirm whether CTX affects the expression of RNA effectors in a time-dependent manner, after CTX treatment, mouse ovaries were collected at different time points. Our results revealed that the mRNA expression levels of YTHDF1 (63, 33, and 21%), YTHDF2 (57, 31, and 15%), YTHDC1 (51, 32, and 27%) and YTHDF3 (64, 31, and 17%) were significantly lower at 2, 4, and 8 weeks, respectively, than the levels of the control group (0 week) (Figures 8A–D). In addition, western blot assay results demonstrated that the protein expression levels of YTHDF1 (56, 35, and 21%) and YTHDF2 (52, 31, and 20%) were reduced gradually at 2, 4, and 8 weeks, respectively, compared to the expression levels of the control group (0 week) (Figure 8E). CTX significantly inhibited the protein expression levels of YTHDC1 and YTHDF3 to 65, 32, and 16%, and to 75, 36, and 14% at 2, 4, and 8 weeks, respectively, relative to the expression levels of the control group (Figure 8F).

As a whole, CTX decreased the mRNA and protein expression levels of RNA effectors in a time-dependent manner.

## Discussion

The senescence and apoptosis of ovarian granulosa cells are important causes of the decline in ovarian reserve (17). Moreover, young patients treated with chemotherapy or radiotherapy may suffer from gonadal damage, permanent ovarian failure and, consequently, a menopausal state with ensuing infertility (18). Cyclophosphamide as an index drug of the alkylating category is a widely used chemotherapeutic and immunosuppressive agent with the greatest gonadotoxic potential impacts on more broadly on aging-related declines and pathologies, such as dormant primordial follicles and on the fraction of growing follicles (preantral/antral) *in vivo* (19). Little is known about the genetics of chemotherapy-related ovarian failure at the RNA methylation level.

RNA methylation is a critical regulator of the cell proliferative state. Its role in cell division is elicited via its impact on the assembly of the mitotic spindle and the nucleolar RNA processing machinery. In this study, we first report that CTX down regulated m^6^A mRNA levels in a concentration- and time-dependent manner (Figure 1). Our previous study showed that the level of m^6^A was higher in human POI disease than in normal people (14). A previous study also revealed that the degrees of absent and decreased follicles were increased by CTX in four stages (primordial follicles, primary follicles, secondary follicles and antral follicles) following the concentration increase (14). m^6^A can be installed by a methyltransferase complex. Here, we presented a number of findings demonstrating the significance of RNA methyltransferases in CTX and ovarian development: (1) CTX increased the gene and protein expression of METTL3, METTL14, ZC3H13, and KIAA1429 in a concentration-dependent manner *in vitro* and *in vivo* (Figures 2, 3); (2) CTX repressed the gene and protein expression of METTL3, METTL14, ZC3H13, and KIAA1429 in a time-dependent manner (Figure 4). Previous studies have shown that deletion of METTL3 in germ cells led to defective spermatogonial differentiation and meiosis (11). Moreover, ZC3H13 depletion impairs self-renewal and triggers mouse embryonic stem cell (mESC) differentiation (20). ZC3H13 plays a critical role in anchoring KIAA1429 in the nucleus to facilitate m^6^A methylation and to regulate mESC self-renewal (21). However, CTX did not change the expression of RNA methyltransferases WTAP and RBM15. One possible reason is that WTAP and RBM15 are regulatory proteins of m^6^A that are dependent on METTL3 and METTL14. A previous study showed that METTL3 levels are critical for WTAP protein homeostasis. Furthermore, the catalytic effect of WTAP on m^6^A is bound by METTL3 and METTL14. Epitranscriptomic mRNA modifications are reversible and are dynamically regulated by RNA demethylases that catalyze the removal of m^6^A from mRNA. In the present study, we found that CTX inhibited the gene and protein expression of FTO in a time- and concentration-dependent manner (Figure 5), but that it did not inhibit ALKBH5. Our previous research found that the mRNA and protein expression level of FTO was lower in patients with POI disease than in normal people (14). In addition, knockdown of FTO decreased the proliferation rate and increased the apoptosis rate (14). RNA effectors of the m^6^A mark preferentially bind to m^6^A and elicit downstream functions by regulation of mRNA splicing and nuclear export. Here, we report a number of findings demonstrating the significance of RNA effectors in CTX and ovarian development: (i) CTX repressed the mRNA and protein expression of YTHDF1, YTHDF2, YTHDC1, and YTHDF3 in a concentration-dependent manner *in vitro* and *in vivo* (Figures 6, 7); (ii) CTX repressed the gene and protein expression of YTHDF1, YTHDF2, YTHDC1, and YTHDF3 in a time-dependent manner (Figure 8). A previous study supported our results that loss of YTHDF2 directly results in the upregulation of the bound transcripts during meiotic and oocyte maturation (21). YTHDC1 deficiency causes massive alternative splicing defects in the processing of pre-mRNA transcripts in the oocyte nucleus (15). YTHDF3 plays critical roles to accelerate the metabolism of m^6^A -modified mRNAs in the cytoplasm and affects translation and decay of methylated mRNAs through cooperation with YTHDF1 and YTHDF2 (22, 23).

In summary, our study is the first to show that CTX inhibits ovarian development by affecting the content of m^6^A that is highly associated with the expression level of RNA methyltransferases, demethylases, and effectors. Our research also demonstrates that CTX promotes m^6^A and methyltransferase levels and represses demethylases and effectors in a time- and concentration-dependent manner. Although the signaling pathways and cellular stresses influence differential distribution patterns of RNA methylation is not yet well-defined, the underlying mechanisms of the effects of CTX on m^6^A levels in the ovary still needs further investigation. Further studies are required to elucidate the functional mechanism in follicle quality that underlies the association between CTX and m^6^A levels. In particular during the period of ovarian aging, the modification in m^6^A regulatory factors need to be explored in the future. The gene accession number is as follows METTL3: Q86U44; METTL14: Q9HCE5; WTAP: Q15007; ZC3H13: Q5T200; KIAA1429: Q69YN4; RBM15: Q96T37; FTO: Q9C0B1; ALKBH5: Q6P6C2; YTHDF1: Q9BYJ9; YTHDF2: Q9Y5A9; YTHDF3: Q7Z739; YTHDC1: Q96MU7.

## Data Availability

All datasets for this study are included in the manuscript and the Supplementary Files.

## Ethics Statement

The use of human ovarian granular cells were in accordance with the relevant guidelines and regulations and the experimental protocols were approved by the Medical Ethics Committee of the Suzhou Hospital Affiliated to Nanjing Medical University. All the patients provided written informed consent prior to participation in this study. Our investigation using experimental animals was conducted on the basis of the Nanjing Medical University Animal Center's specific guidelines and standards.

## Author Contributions

CD performed cellular and molecular assay *in vivo* and *in vitro*. QZ contributed to hGCs collection, purification, and culture. CD and BH established different level ovarian aging mice model by CTX. WW contributed to recruit patients. BH and HL planned the experiments and wrote the manuscript.

### Conflict of Interest Statement

The authors declare that the research was conducted in the absence of any commercial or financial relationships that could be construed as a potential conflict of interest.
