# Supplementary material for: Cyclophosphamide Regulates N6-Methyladenosine and m6A RNA Enzyme Levels in Human Granulosa Cells and in Ovaries of a Premature Ovarian Aging Mouse Model
Source: Front Endocrinol (Lausanne). 2019 Jun 27;10:415. doi: 10.3389/fendo.2019.00415 (PMC6610338; doi:10.3389/fendo.2019.00415)
Supplement: Supplementary file 1 [file Table_1.DOC]

**Supplemental Table 1 Designations, sequences, and the sizes of real-time PCR amplicons**

**H=Human; M=Mouse.**

| **Name** | **Sequence from 5'-3'** | **Size (bp)** |
| --- | --- | --- |
| ALKBH5 (H) Fw | AGGGGAAGCGTGACTGTGC | 123 |
| ALKBH5 (H) Rev | GGGTGCATCTAATCTTGTCTTCC |
| FTO (H) Fw | CTTCACCAAGGAGACTGCTATTTC | 129 |
| FTO (H) Rev | CAAGGTTCCTGTTGAGCACTCTG |
| WTAP (H) Fw | TGTGCTGTGTAAGGGCATTCGTACTCATGC | 102 |
| WTAP (H) Rev | ACTGGGCAAACTTGGCAGTCATAAACCCAC |
| METTL3 (H) Fw | CCAGGGGTCATTTTCCGGTT | 150 |
| METTL3 (H) Rev | GATAGAGCTCCACGTGTCCG |
| METTL14 (H) Fw | ACCTTGGAAGAGTGTGTTTACGA | 142 |
| METTL14 (H) Rev | TGTGAGCCAGCCTTTGTTCT |
| ZC3H13 (H) Fw | GAAGCTGCCTGGGTCTACAG | 224 |
| ZC3H13 (H) Rev | CCAGTTACGGCACTGTGTCT |
| KIAA1429 (H) Fw | GAATTCGCCCTTCTGAGTCG | 218 |
| KIAA1429 (H) Rev | TGGGGGTATGACTCGGACTT |
| RBM15 (H) Fw | TGAGTTCTCCCAGCAGTTCC | 262 |
| RBM15 (H) Rev | CTCCACATAAGCCACAACTTCTC |
| YTHDF1 (H) Fw | AACAATGAGGGCGAACCAGT | 91 |
| YTHDF1 (H) Rev | GACACACTGGAGCTGACCAA |
| YTHDF2 (H) Fw | TAGCCAACTGCGACACATTC | 212 |
| YTHDF2 (H)Rev | CACGACCTTGACGTTCCTTT |
| YTHDF3 (H) Fw | TGACAACAAACCGGTTACCA | 179 |
| YTHDF3 (H) Rev | TGTTTCTATTTCTCTCCCTACGC |
| YTHDC1 (H) Fw | TCATCTTCCGTTCGTGCTGT | 147 |
| YTHDC1 (H) Rev | TACAGGGAGCGTGGACCATA |
| GAPDH (H) Fw | GAAGGTCGGAGTCAACGGATTT | 223 |
| GAPDH (H) Rev | CTGGAAGATGGTGATGGGATTTC |
| ALKBH5 (M) Fw | CAGTGGGTATGCTGCTGATG | 186 |
| ALKBH5 (M) Rev | GGGTCTCTGGTGTTTCCTGA |
| FTO (M) Fw | GGCTGTGGAAGAAGATGGAG | 190 |
| FTO (M) Rev | TGCTGTGCTGGTAGAGTTCG |
| WTAP (M) Fw | GCCCCAACGTTTAAGTGCAG | 106 |
| WTAP (M) Fw | AGCATTCGACACTTCGCCAT |
| METTL3 (M) Fw | ATCTTGGCTCTATCCGGCTG | 147 |
| METTL3 (M) Fw | GATAGAGCTCCACGTGTCCG |
| METTL14 (M) Fw | TGGATTTGCATTTTGGCGGG | 152 |
| METTL14 (M) Fw | ATGCTATCCGCACTCTCAGC |
| ZC3H13 (M) Fw | TGTTTCAGACGGGACTCTGC | 78 |
| ZC3H13 (M) Fw | GTTTGTGTACGCCTGCTTCA |
| KIAA1429 (M) Fw | CAGTCGTCGGGAAGGAACAA | 149 |
| KIAA1429 (M) Fw | TAGGGCGGTAACCCGTAGAA |
| RBM15 (M) Fw | GCAGCATCCTCTCCCAAACT | 98 |
| RBM15 (M) Fw | AGGTCACCCTGCAACAGATG |
| YTHDF1 (M) Fw | CTGCAGTTAAGACGGTGGGT | 123 |
| YTHDF1 (M) Rev | TAGCAATGGCTGCCCATGAA |
| YTHDF2 (M) Fw | AGGCGGGTTCTGGATCTACT | 216 |
| YTHDF2 (M)Rev | GATAGGCGGCATCCAGTCTC |
| YTHDF3 (M) Fw | CAGAGACCTAAAGGGCAAGGA | 239 |
| YTHDF3 (M) Rev | CATGCTGCTTCCCCAAGAGA |
| YTHDC1 (M) Fw | CGTAGGAAGCTGAGTGGAGC | 188 |
| YTHDC1 (M) Rev | TCCCCATCTTTCTCCTCCCG |
| GAPDH (M) Fw | TTCCAGTATGACTCTACCCACGGCA | 137 |
| GAPDH (M) Rev | GCACCAGCATCACCCCATTTG |
