# Supplementary material for: Cyclophosphamide Regulates N6-Methyladenosine and m6A RNA Enzyme Levels in Human Granulosa Cells and in Ovaries of a Premature Ovarian Aging Mouse Model
Source: Front Endocrinol (Lausanne). 2019 Jun 27;10:415. doi: 10.3389/fendo.2019.00415 (PMC6610338; doi:10.3389/fendo.2019.00415)
Supplement: Supplementary file 2 [file Table_2.DOC]

**Supplemental Table 2 Information of patient hGCs**

|  | Age | E2  (pg/ml) | AMH  (ng/ml) | FSH  (IU/ml) | Antral follicles | Smoking | Alcohol |
| --- | --- | --- | --- | --- | --- | --- | --- |
| N1 | 30 | 49 | 4.33 | 7.61 | 18 | No | No |
| N2 | 32 | 55 | 5.12 | 5.97 | 21 | No | No |
| N3 | 30 | 59 | 3.89 | 6.54 | 20 | No | No |
| N4 | 29 | 57 | 3.91 | 7.52 | 21 | No | No |
| N5 | 27 | 49 | 4.79 | 4.36 | 17 | No | No |
| N6 | 26 | 51 | 5.12 | 6.50 | 15 | No | No |
| N7 | 29 | 52 | 3.77 | 7.71 | 19 | No | No |
| N9 | 33 | 46 | 3.89 | 5.45 | 17 | No | No |
| N10 | 34 | 47 | 4.62 | 6.30 | 20 | No | No |
| N11 | 31 | 57 | 5.17 | 6.12 | 21 | No | No |
| N12 | 22 | 54 | 5.25 | 5.89 | 19 | No | No |
| N13 | 25 | 50 | 4.58 | 6.73 | 19 | No | No |
| N15 | 23 | 42 | 3.26 | 7.25 | 21 | No | No |
| N16 | 27 | 57 | 4.66 | 7.50 | 22 | No | No |
| N17 | 29 | 49 | 3.75 | 5.46 | 20 | No | No |
| N18 | 31 | 48 | 4.71 | 6.49 | 16 | No | No |

**N=Normal**

Culture plates with six-well were used for hGCs, 2×10^6^ cells were planted in very well. After hGCs were cultured in medium up to 12 days, the subsequent experiments were executed. CTX (Sigma, USA) was used at different doses (20, 40, and 60 μg/ml respectively). All experiments were performed three times for biological duplication. The purpose of treatment is aim to carry out oocyte retrieval, and then the follicular fluid was collected. All control group people (tubal occlusion) were recruited from Suzhou hospital affiliated to Nanjing medical university.
